# Supplementary material for: A rapid realist review of universal interventions to promote inclusivity and acceptance of diverse sexual and gender identities in schools
Source: Nat Hum Behav. 2023 Feb 13;7(4):556–67. doi: 10.1038/s41562-023-01521-z (PMC10129865; doi:10.1038/s41562-023-01521-z)
Supplement: Supplementary file 2 — Reporting Summary [file 41562_2023_1521_MOESM2_ESM.pdf]

nature portfolio

Corresponding author(s): Gemma Lewis

Last updated by author(s): 05/01/2023

Reporting Summary

Nature Portfolio wishes to improve the reproducibility of the work that we publish. This form provides structure for consistency and transparency in reporting. For further information on Nature Portfolio policies, see our Editorial Policies and the Editorial Policy Checklist.

Statistics

For all statistical analyses, confirm that the following items are present in the figure legend, table legend, main text, or Methods section.

n/a	Confirmed	
☒	☐	The exact sample size (*n*) for each experimental group/condition, given as a discrete number and unit of measurement
☒	☐	A statement on whether measurements were taken from distinct samples or whether the same sample was measured repeatedly
☒	☐	The statistical test(s) used AND whether they are one- or two-sided<br>*Only common tests should be described solely by name; describe more complex techniques in the Methods section.*
☒	☐	A description of all covariates tested
☒	☐	A description of any assumptions or corrections, such as tests of normality and adjustment for multiple comparisons
☒	☐	A full description of the statistical parameters including central tendency (e.g. means) or other basic estimates (e.g. regression coefficient) AND variation (e.g. standard deviation) or associated estimates of uncertainty (e.g. confidence intervals)
☒	☐	For null hypothesis testing, the test statistic (e.g. *F*, *t*, *r*) with confidence intervals, effect sizes, degrees of freedom and *P* value noted<br>*Give P values as exact values whenever suitable.*
☒	☐	For Bayesian analysis, information on the choice of priors and Markov chain Monte Carlo settings
☒	☐	For hierarchical and complex designs, identification of the appropriate level for tests and full reporting of outcomes
☒	☐	Estimates of effect sizes (e.g. Cohen's *d*, Pearson's *r*), indicating how they were calculated

Our web collection on statistics for biologists contains articles on many of the points above.

Software and code

Policy information about availability of computer code

Data collection	As this study was a review, primary data were not collected. The software Rayyan was used for the collection and analysis of data from existing sources (published and unpublished studies and sources).
Data analysis	No statistical code was used; Rayyan does not involve coding and no primary data were analysed because this study was a review of existing literature.

For manuscripts utilizing custom algorithms or software that are central to the research but not yet described in published literature, software must be made available to editors and reviewers. We strongly encourage code deposition in a community repository (e.g. GitHub). See the Nature Portfolio guidelines for submitting code & software for further information.

Data

Policy information about availability of data

All manuscripts must include a data availability statement. This statement should provide the following information, where applicable:

- Accession codes, unique identifiers, or web links for publicly available datasets
- A description of any restrictions on data availability
- For clinical datasets or third party data, please ensure that the statement adheres to our policy

The data that support the findings of this study are included in Supplementary Tables 1, 2 and 3 and any further details are available from the corresponding author upon reasonable request. All data used in this study are available in the individual studies which constitute the review. Submission to a public repository is not applicable. We conducted an exploratory scoping search using Google Scholar to identify key sources and reviews and develop an initial programme theory. For the main search, we searched PubMed, PsycINFO and Web of Science (search terms in Supplementary Table 2). We consulted experts, the YPAG, and relevant organisations to identify grey literature. A Call for Evidence was disseminated via Twitter to invite schools, organisations, and young people to submit evidence.

Field-specific reporting

Please select the one below that is the best fit for your research. If you are not sure, read the appropriate sections before making your selection.

☒ Life sciences ☐ Behavioural & social sciences ☐ Ecological, evolutionary & environmental sciences

For a reference copy of the document with all sections, see nature.com/documents/nr-reporting-summary-flat.pdf

Life sciences study design

All studies must disclose on these points even when the disclosure is negative.

Sample size	As this was a rapid realist review (with no analysis of aggregate data or meta-analysis), there was no single sample size
Data exclusions	As this study was a review there were not data exclusions other than the exclusion criteria for the review itself
Replication	Replication was not applicable because this study was a Rapid Realist Review so replication was not required.
Randomization	Randomization was not required because this study was a Rapid Realist Review (rather than a trial or any other kind of experimental design).
Blinding	Blinding was not required because this study was a Rapid Realist Review (rather than a trial or any other kind of experimental design).

Reporting for specific materials, systems and methods

We require information from authors about some types of materials, experimental systems and methods used in many studies. Here, indicate whether each material, system or method listed is relevant to your study. If you are not sure if a list item applies to your research, read the appropriate section before selecting a response.

Materials & experimental systems

n/a	Involved in the study	
☒	☐	Antibodies
☒	☐	Eukaryotic cell lines
☒	☐	Palaeontology and archaeology
☒	☐	Animals and other organisms
☒	☐	Human research participants
☒	☐	Clinical data
☒	☐	Dual use research of concern

Methods

n/a	Involved in the study	
☒	☐	ChIP-seq
☒	☐	Flow cytometry
☒	☐	MRI-based neuroimaging
